# Supplementary material for: Electromagnetic Wavefront Engineering by Switchable and Multifunctional Kirigami Metasurfaces
Source: Nanomaterials (Basel). 2025 Jan 2;15(1):61. doi: 10.3390/nano15010061 (PMC11722745; doi:10.3390/nano15010061)
Supplement: Supplementary file 1 [file nanomaterials-15-00061-s001.zip › nanomaterials-3342305-supplementary.pdf]

Supplementary Materials

Electromagnetic Wavefront Engineering by Switchable and Multifunctional Kirigami Metasurfaces

Yingying Wang [1,2,†], Yang Shi [1,2,†], Liangwei Li [1,2], Zhiyan Zhu [1,2], Muhan Liu [1,2], Xiangyu Jin [1,2], Haodong Li [3,4], Guobang Jiang [5,6], Jizhai Cui [5,6], Shaojie Ma [1], Qiong He [3,4,7], Lei Zhou [3,4,7,*] and Shulin Sun [1,2,*]

[1] Shanghai Engineering Research Centre of Ultra Precision Optical Manufacturing, Department of Optical Science and Engineering, School of Information Science and Technology, Fudan University, Shanghai 200433, China; 21210720013@m.fudan.edu.cn (Y.W.); 22110720011@m.fudan.edu.cn (Y.S.); 21210720008@m.fudan.edu.cn (L.L.); 21110720021@m.fudan.edu.cn (Z.Z.); 21110720013@m.fudan.edu.cn (M.L.); 22210720009@m.fudan.edu.cn (X.J.); shaojiema@fudan.edu.cn (S.M.)
[2] Yiwu Research Institute, Fudan University, Chengbei Road, Yiwu 322000, China
[3] State Key Laboratory of Surface Physics (Ministry of Education), Fudan University, Shanghai 200433, China; 19110190007@fudan.edu.cn (H.L.); qionghe@fudan.edu.cn (Q.H.)
[4] Key Laboratory of Micro and Nano Photonic Structures (Ministry of Education), Fudan University, Shanghai 200433, China
[5] Department of Materials Science and State Key Laboratory of Molecular Engineering of Polymers, Fudan University, Shanghai 200438, China; 21110300010@m.fudan.edu.cn (G.J.); jzcui@fudan.edu.cn (J.C.)
[6] International Institute of Intelligent Nanorobots and Nanosystems, Fudan University, Shanghai 200438, China
[7] Collaborative Innovation Center of Advanced Microstructures, Nanjing 210093, China
* Correspondence: phzhou@fudan.edu.cn (L.Z.); sls@fudan.edu.cn (S.S.)
† These authors contributed equally to this work.

Section A. Design and fabrication of RS kirigami transformation substrates

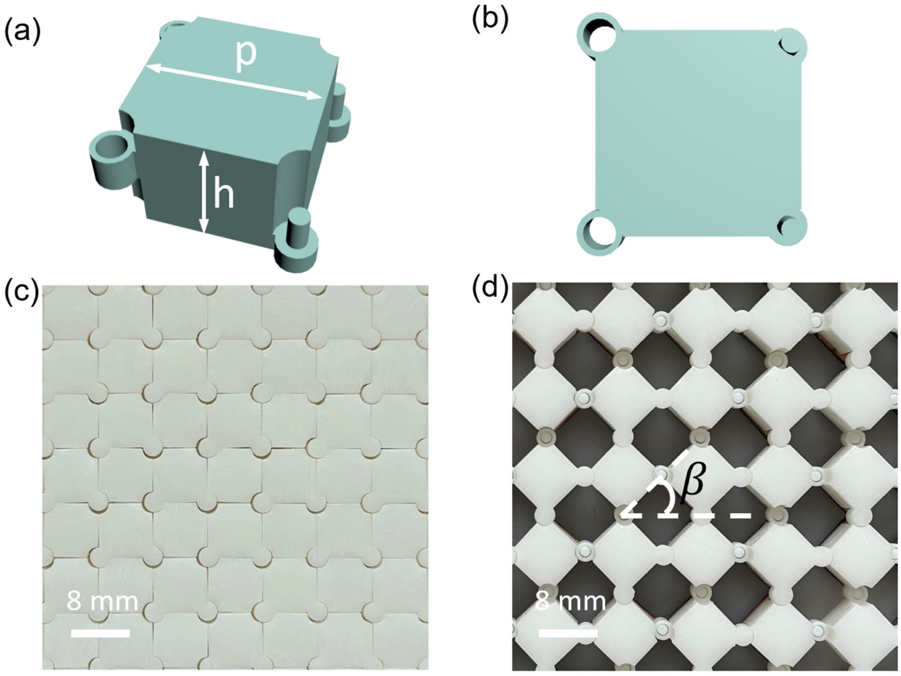

Figure S1. Design and fabrication of the RS kirigami transformation substrates. (**a**, **b**) Schematics of the designed unit cell of a RS transformable substrate in the perspective view and top view, respectively. Here, the lattice constant and the thickness of the unit cell is p = 8 mm and h = 10 mm, respectively. (**c**, **d**) Sample images of the assembled transformable substrate in the initial (β = 0°) and the transformed (β = 45°) states, respectively.

Section B. Design parameters of the metasurface shown in Figure 3

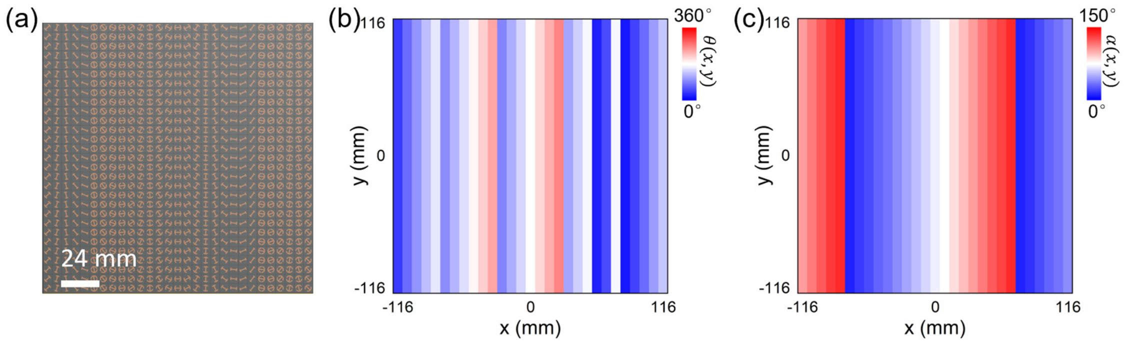

Figure S2. Detailed information of the multiple-beam meta-reflector shown in Figure 3. (**a**) Image of the fabricated metasurface. (**b**, **c**) The spatial distributions of orientation angle $\theta(x, y)$ and opening angle $\alpha(x, y)$ of the metasurface described in (**a**).

Section C. Comparison of the beam bending effect by the kirigami metasurface with the ideally discontinuous and continuous phase profiles

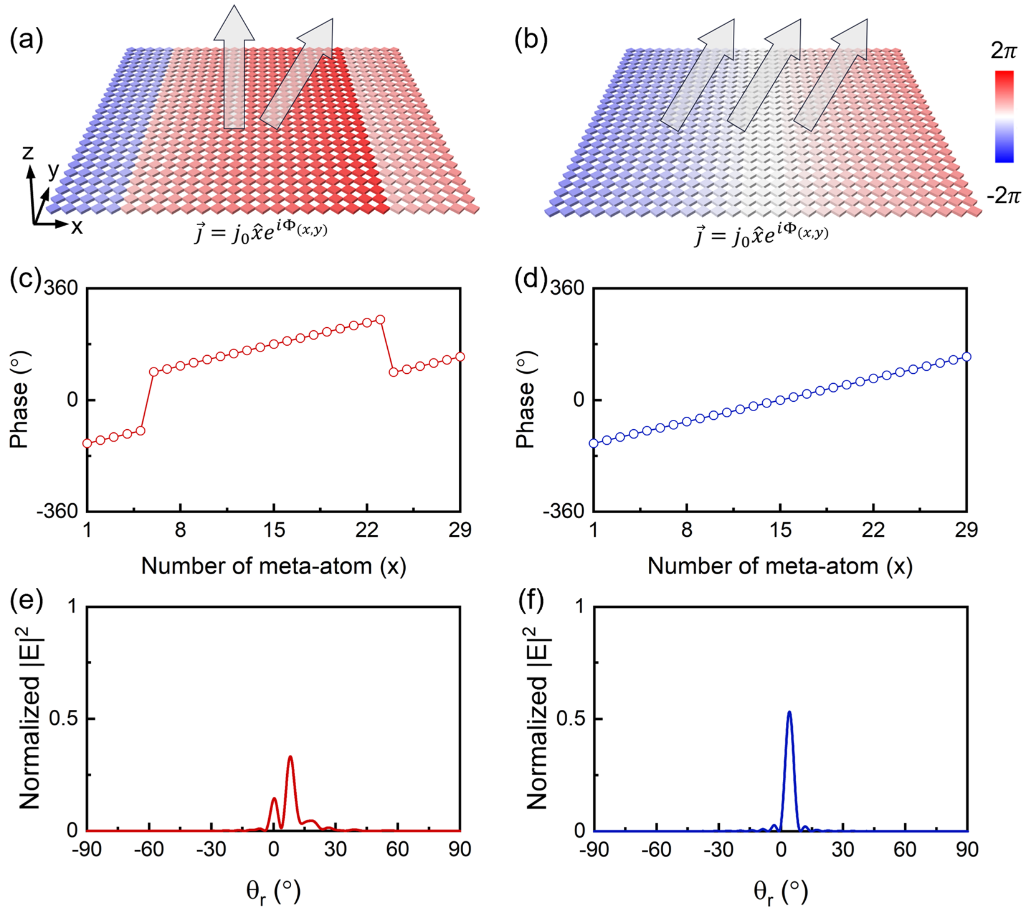

Figure S3. A surface current model for demonstrating anomalous reflection effect by the kirigami metasurface with discontinuous and continuous phase profiles (corresponding to Figure 3). (**a**, **b**) Radiation effect of an ideal surface current source $\vec{J} = j_0\hat{x}e^{i\phi(x,y)}$ excited on the kirigami metasurface with the discontinuous and continuous phase profiles of $\phi(x, y)$ shown in (**c**) and (**d**), respectively. (**e**, **f**) Calculated normalized scattering field distributions as a function of the reflection angle θ_r for the two metasurfaces depicted in (a) and (b) at the frequency of 10 GHz. It is noted that, except for the anomalous reflection beam, another normal reflection signal along $\theta_r = 0°$ is also generated in (e), caused by the discontinuity of the phase profile. This phenomenon is similar to the specular normal mode generation of the kirigami metasurface with the discontinuous resonant phase distribution at the state of β = 45° (see Figure 3c,f,i,l). Conversely, the ideal metasurface that can possess a linear resonant phase without any discontinuity will generate a single non-specular deflection beam as shown in (f).

Section D. Broadband performance of the kirigami metasurface in Figure 3

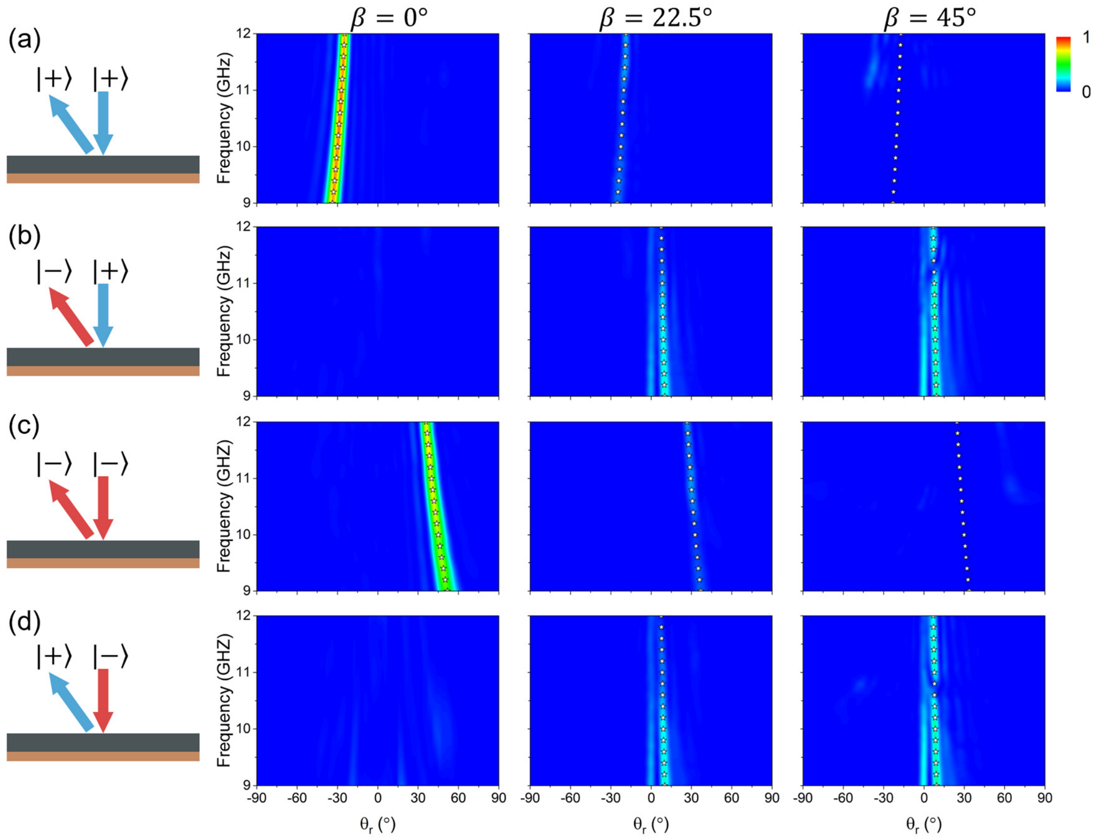

Figure S4. Numerical verification of the broadband switchableand multifunctional beam steering by the RS kirigami metasurface shown in Figure 3. (**a-d**) Simulated normalized scattering field intensities with LCP (|+⟩) or RCP (|−⟩) at the reflection side as the functions of working frequency and reflection angle for the kirigami metasurface in three representative transformation states (β = 0°, 22.5°, 45°) illuminated by the normally incident LCP or RCP wave. Here, open stars represent theoretical predictions according to the generalized Snell's law.

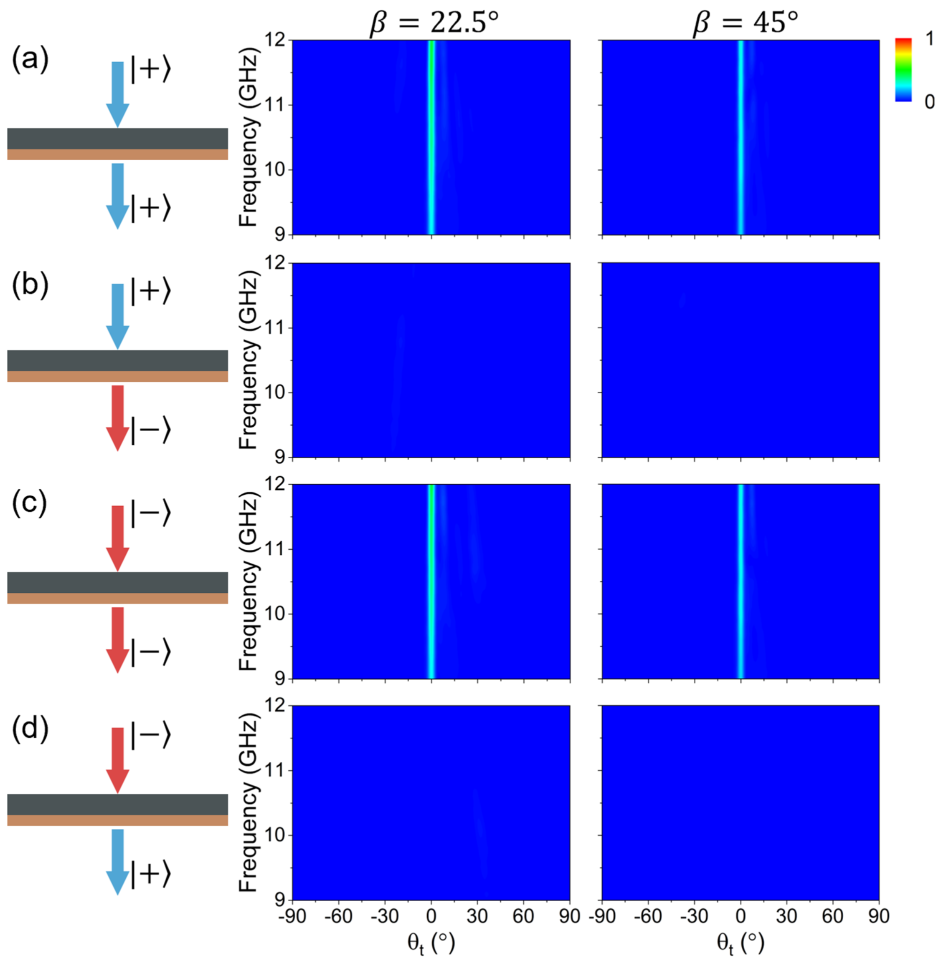

Figure S5. Simulated normalized scattering field intensities at the transmission side of kirigami metasurface in β = 22.5°and β = 45°states. (**a-d**) Normalized scattering electric field intensities with LCP (| +⟩) or RCP (| −⟩) as the functions of working frequency and transmission angle θ_t for the kirigami metasurfaces shown in Figure 3 in β = 22.5°and β = 45°states illuminated by normally incident RCP or RCP wave. Since such reflective meta-atoms have a continuous metal mirror as the bottom layer, which will not provide any transmission signal (including amplitude and phase). Therefore, the input wave can only penetrate the transformed metasurface (β ≠ 0°) through the air holes appearing between the adjacent meta-atoms, generating the normal co-polarization transmission signals along $\theta_t = 0°$

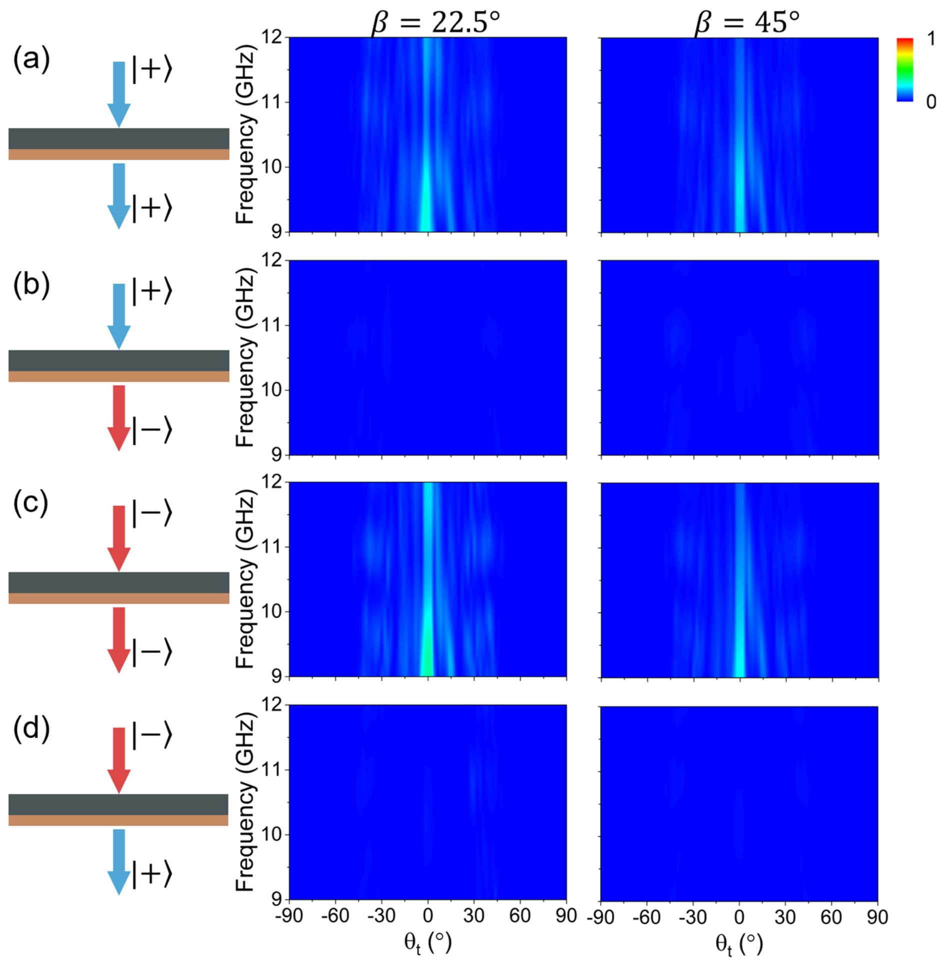

Figure S6. Measured normalized scattering field intensities at the transmission side of kirigami metasurface in β = 22.5°and β = 45°states. (**a-d**) Normalized scattering field intensities as the functions of working frequency and transmission angle for the kirigami metasurfaces shown in Figure 3 in β = 22.5°and β = 45°states. In our experiments, the device is illuminated normally by an LCP (| +⟩)or RCP (| −⟩) horn antenna and received by another LCP or RCP horn antenna. The measured results also demonstrate that part of incident waves will penetrate the transformed metasurface (β ≠ 0°) through the air holes appearing between the adjacent meta-atoms as the normal co-polarization transmission mode along θ_t = 0°, showing good agreement with the simulated results.

Section E. Tunable functionalities of the kirigami metasurface shown in Figure 3

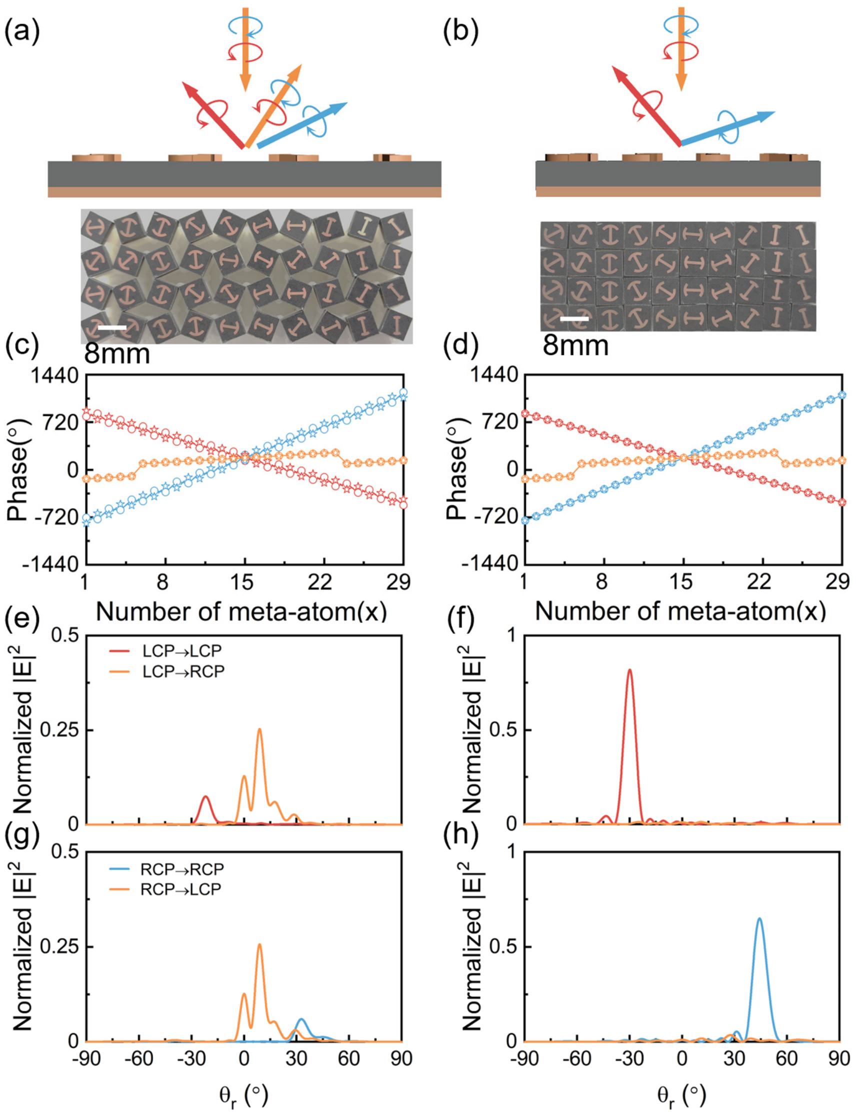

Figure S7. Characterization of the switchable multiple-beam meta-reflector in β = 67.5°and β = 90°states. (**a**, **b**) Schematics and sample images of the kirigami metasurface in β = 67.5°and β = 90° states illuminated by LCP and RCP waves. (**c**, **d**) The reflection phase profiles along the x direction of kirigami metasurfaces in two states illuminated by the LCP and RCP wave at the frequency of 10 GHz. (e, f) Simulated normalized scattering field angular distributions of the kirigami metasurfaces in two states illuminated by LCP wave at the frequency of 10 GHz. (**g**, **h**) Simulated normalized scattering field angular distributions of the kirigami metasurfaces in two states illuminated by RCP wave at the frequency of 10 GHz.

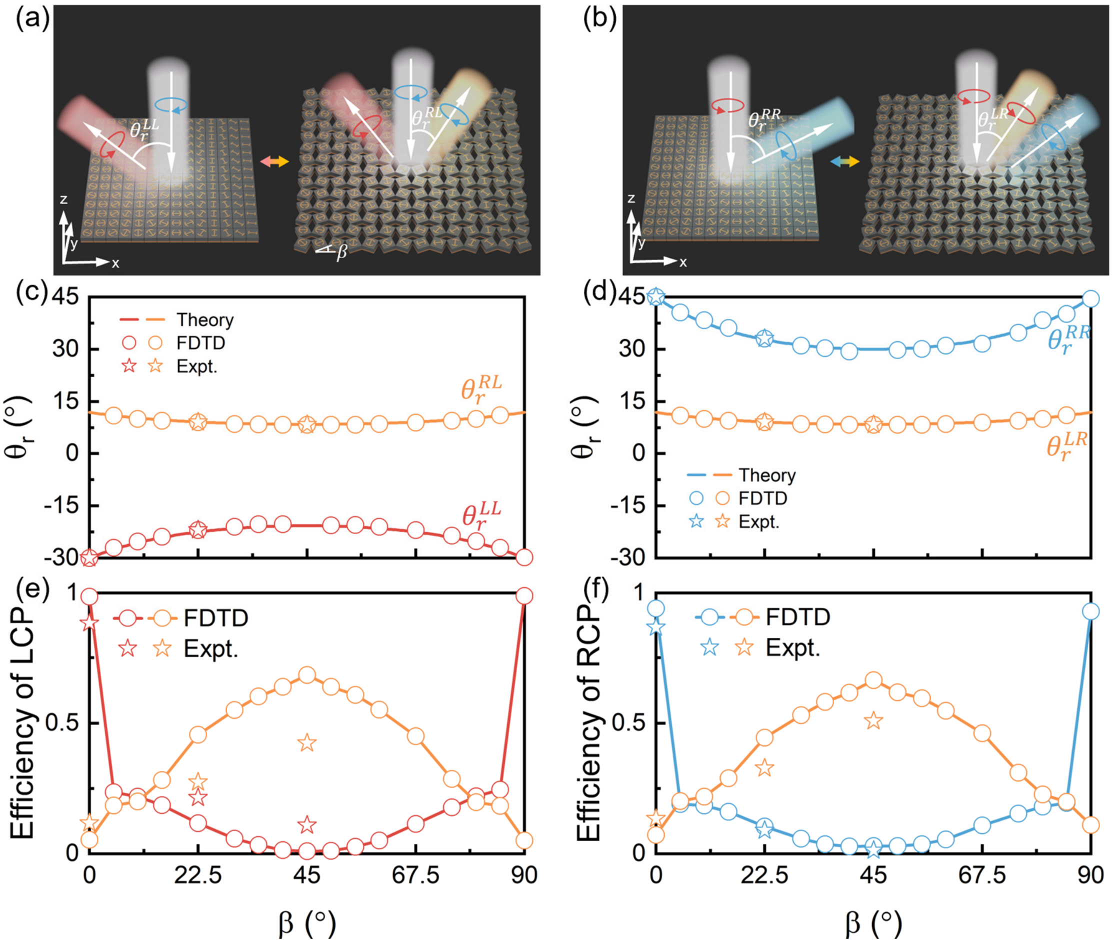

Figure S8. Verification of tunable anomalous beam deflection effect by the kirigami metasurface shown in Figure 3. (**a**, **b**) Schematics of switchable beam deflection by RS kirigami metasurface in different states illuminated by LCP and RCP wave, respectively. The red and blue beams represent the anomalous mode (target 1) with reflection angle θ_r^{LL} under LCP wave incidence and the anomalous mode (target 2) with reflection angle θ_r^{RR} under RCP wave incidence, respectively. Besides, the orange beam represents the normal mode (target 3) with reflection angle θ_r^{RL} or θ_r^{LR}. (**c**, **d**) Modulations of the reflection angles for anomalous (target 1 and 2) and normal (target 3) reflection beams versus β under LCP and RCP wave incidence. It is noted that both the full-wave simulations and microwave experiments agree well with theoretical predictions. Since the normal mode is determined by the spin-independent resonant phase of the metasurface, the reflection angles of the normal mode under LCP and RCP wave illuminations are equal, i.e., $\theta_r^{RL} = \theta_r^{LR}$. (**e**, **f**) Simulated and measured efficiencies of the three

reflection beams generated by the kirigami metasurface under the illumination of LCP and RCP waves. Here, the efficiencies are obtained by dividing the integrated energy for the desired reflection modes by the integrated power of the input beam. As discussed in the main text, because the effective PCR of composite meta-atoms first decreases from 100% to nearly 0 and then increases back to 100% during the transformation process, the anomalous (normal) mode first is dominated (suppressed) and then suppressed (dominated). Here, the frequency is fixed at 10 GHz.

Section F. Design parameters of the kirigami metasurface corresponding to Figure 5

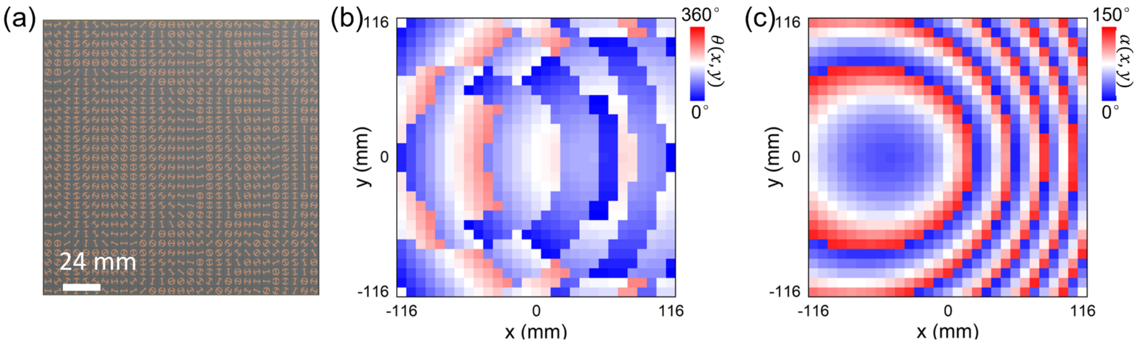

Figure S9. Image and geometric parameters of the kirigami metasurface shown in Figure 5 for achieving switchable complex wavefront engineering. (**a**) Image of the kirigami metasurface corresponding to Figure 5. (**b**, **c**) The distribution of orientation angle $\theta(x, y)$ and opening angle $\alpha(x, y)$ of the kirigami metasurface described in (a).

Section G. Numerical demonstrations of the straight focusing and tilted focusing by the kirigami metasurface in Figure 6

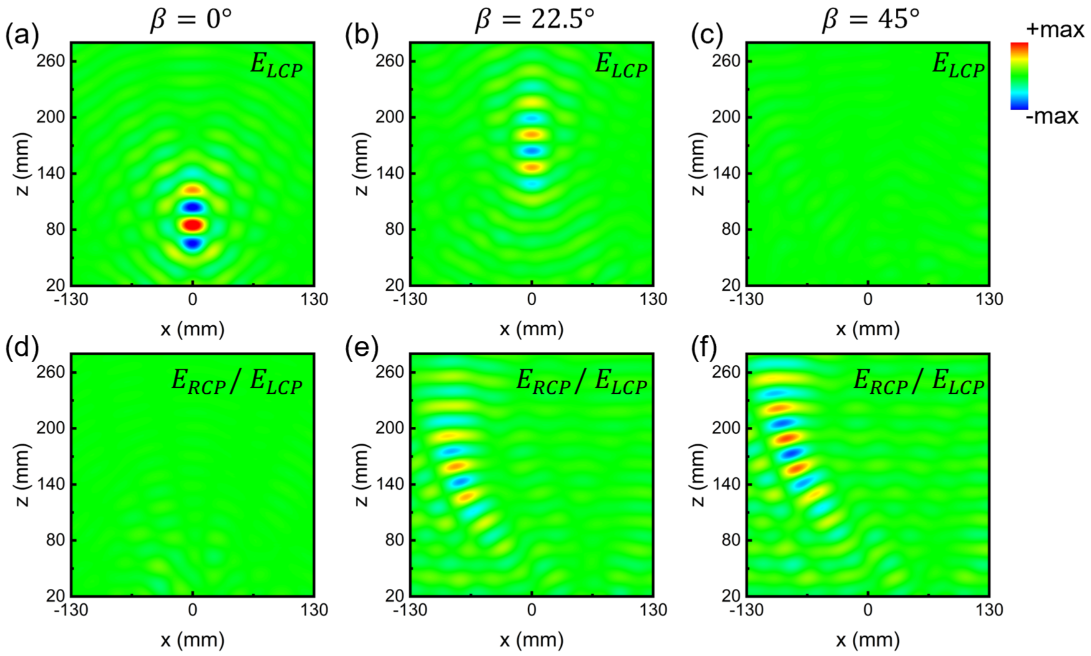

Figure S10. Numerical characterization of the straight focusing and tilted focusing effect by the kirigami metasurface in Figure 6 in β = 0°, β = 22.5° and β = 45° states. (**a-c**) Simulated electric field distributions with LCP (E_{LCP}) in the XOZ plane for kirigami metasurfaces in three different states under the illumination of LCP wave. (**d-f**) Simulated electric field intensity distributions E_{LCP} or E_{RCP} in the XOZ plane of the kirigami metasurfaces in three different states under the illumination of LCP or RCP wave.
